# Metabolic Profiling in Patients with Pneumonia on Intensive Care

**DOI:** 10.1016/j.ebiom.2017.03.034

**Published:** 2017-03-29

**Authors:** David Antcliffe, Beatriz Jiménez, Kirill Veselkov, Elaine Holmes, Anthony C. Gordon

**Affiliations:** a Section of Anaesthetics, Pain Medicine and Intensive Care, Department of Surgery and Cancer, Faculty of Medicine, Imperial College London, UK; b Division of Computational and Systems Medicine, Department of Surgery and Cancer, Faculty of Medicine, Imperial College London, UK

**Keywords:** Metabonomics, Pneumonia, Ventilatior associated pneumonia (VAP), Intensive care, Brain injury, Ventilation

## Abstract

Clinical features and investigations lack predictive value when diagnosing pneumonia, especially when patients are ventilated and when patients develop ventilator associated pneumonia (VAP). New tools to aid diagnosis are important to improve outcomes. This pilot study examines the potential for metabolic profiling to aid the diagnosis in critical care.

In this prospective observational study ventilated patients with brain injuries or pneumonia were recruited in the intensive care unit and serum samples were collected soon after the start of ventilation. Metabolic profiles were produced using 1D ^1^H NMR spectra. Metabolic data were compared using multivariate statistical techniques including Principal Component Analysis (PCA) and Orthogonal Partial Least Squares Discriminant Analysis (OPLS-DA).

We recruited 15 patients with pneumonia and 26 with brain injuries, seven of whom went on to develop VAP. Comparison of metabolic profiles using OPLS-DA differentiated those with pneumonia from those with brain injuries (*R*^2^*Y* = 0.91, *Q*^2^*Y* = 0.28, *p* = 0.02) and those with VAP from those without (*R*^2^*Y* = 0.94, *Q*^2^*Y* = 0.27, *p* = 0.05). Metabolites that differentiated patients with pneumonia included lipid species, amino acids and glycoproteins.

Metabolic profiling shows promise to aid in the diagnosis of pneumonia in ventilated patients and may allow a more timely diagnosis and better use of antibiotics.

## Highlights

• Metabolic profiling of serum from ventilated patients shows potential to aid in the diagnosis of pneumonia and ventilator associated pneumonia.
• Lipid species were particularly important in the discrimination of pneumonia and ventilator associated pneumonia from those with brain injuries.
• Metabolites including glycoproteins, amino acids and phospholipids also aided in discrimination.

Pneumonia is a common problem in patients requiring breathing support in Intensive Care Units (ICU). When pneumonia develops patients take longer to get back to full health and in some cases may die. Current diagnostic tests are not always helpful.

We measured an array of molecules, metabolites, in the blood of patients in ICU to compare those with pneumonia to those with brain injuries. We found that substances including lipids, amino acids and glycoproteins showed some ability to differentiate patients with pneumonia from other critically unwell patients. This technique, used with clinical data, may help improve the diagnosis of pneumonia.

## 1 Introduction

Pneumonia is a frequent cause for admission to the Intensive Care Unit (ICU) and ventilator associated pneumonia (VAP) is a common complication in patients requiring mechanical ventilation, occurring in 8–28% of such patients (Chastre and Fagon, 2002). VAP is associated with increased mortality, longer intensive care unit and hospital stays, and increased healthcare costs (Chastre and Fagon, 2002).

Current diagnostic techniques are limited. Clinical features (Fabregas et al., 1999), radiological findings (Fabregas et al., 1999, Wunderink et al., 1992) and laboratory tests (Marquette et al., 1995, Fabregas et al., 1999) lack sensitivity and specificity. Biomarkers including C-reactive protein (CRP) (Povoa et al., 2005), procalcitonin (Kibe et al., 2011), and soluble triggering receptor expressed on myeloid cells (sTREM-1) (Oudhuis et al., 2009) have either failed to show strong clinical benefit or specificity for pneumonia. A new diagnostic technique is required to allow patients with VAP to be differentiated from those without, allowing a more targeted antibiotic strategy.

Metabonomics is the quantitative measurement over time of the metabolic responses of an individual or population to disease, drug treatment or intervention (Holmes et al., 2008) and provides a “top-down” integrated overview of the biochemistry in a complex system. Little work has been done characterizing pneumonia using metabonomic techniques. A few small studies have been performed including a study exploring pneumonia in Gambian children (Laiakis et al., 2010), and another looking at metabolic profiling of urine in patients with *Streptococcus pneumoniae* pneumonia (Slupsky et al., 2009b). Work within critical care has focused on outcomes of patients with community acquired pneumonia (CAP) (Seymour et al., 2013) and the ability of metabonomics to differentiate CAP as a cause for sepsis (Neugebauer et al., 2016). Despite the identification of metabolites associated with pneumonia, none have so far been used clinically as diagnostic tests.

No work has been carried out using metabonomic methods focusing specifically on VAP and limited work has been done looking at methods for differentiating patients with pneumonia from similar critically unwell patients. Most studies have used healthy volunteers as controls, this approach is limited as healthy volunteers are likely to be metabolically very different to severely ill patients. In this study we compare ventilated patients with pneumonia with a control group of patients similarly ventilated to give a more realistic background on which to develop a diagnostic for pneumonia.

## 2 Methods

### 2.1 Study Participants

Patients were recruited following written, informed assent from Imperial College Healthcare NHS Trust, London, in accordance with independent research ethics committee approval (North London REC 10/H0709/77) and conforming to the standards indicated by the Declaration of Helsinki, between December 2011 and December 2013. Patients who had two of the four criteria of the systemic inflammatory response syndrome (Bone et al., 1992) and were expected to require mechanical ventilation for ≥ 48 h were eligible for inclusion. Two groups were enrolled; firstly patients who had a primary diagnosis of brain injury, including subarachnoid hemorrhage, cerebrovascular accident, isolated head injury, status epilepticus or primary brain tumor, with no evidence of pneumonia. The second group were those who had pneumonia at the time of initiation of ventilation. Diagnosis was based initially on the opinion of the treating physician and then further refined by the application of the clinical pulmonary infection score (CPIS) (Pugin et al., 1991), Fig. 1. A brain injury cohort was selected as the control group as this patient group is critically unwell and often requires a period of prolonged ventilation, without having infection at the point of enrolment, thus requiring a similar degree of intensive care support to the pneumonia patients. Also this group has a significant risk of developing VAP providing a chance of being able to prospectively acquire samples from patients developing VAP without the confounding of other infection.

Enrolment occurred within the first 48 h of ICU admission, as soon as written consent could be obtained from a personal or professional consultee. Blood samples were collected as soon after enrolment as possible, all were taken within the first 72 h of ventilation with an average time to the first sample of 43 h, and then at 48 h intervals until either the patient left the ICU or four samples had been collected. Serum was separated immediately by allowing whole blood to clot on ice for 30 min. The serum fraction was isolated by centrifugation for 10–15 min and then stored at − 80 °C prior to batch analysis.

**Fig. 1:**
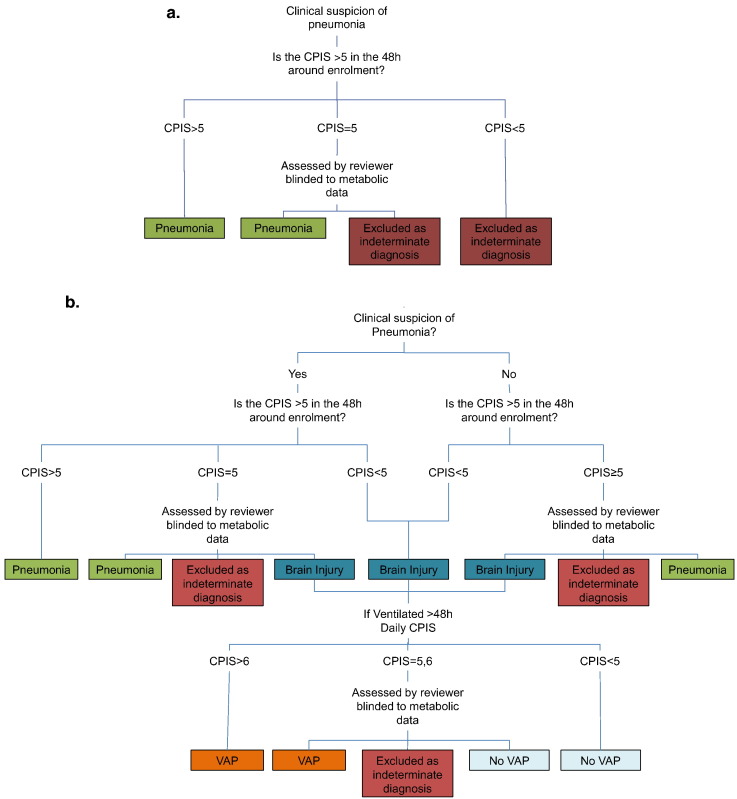
Diagnostic pathway for a) patients admitted with pneumonia (*n* = 15) and b) patients admitted with brain injury (*n* = 26) when enrolled into the study.

Patients with brain injuries were reviewed daily and clinical, routine laboratory and radiological data were collected from the clinical notes. A diagnosis of VAP was made, prospectively, in patients who had been ventilated for > 48 h in whom the CPIS was > 6. Not all components of the CPIS score were routinely measured at our institution, specifically Gram staining of tracheal secretions and the measurement of circulating band forms, so patients with borderline scores were assessed by an independent clinician blinded to the metabonomics results and classified as pneumonia, VAP or no VAP, Fig. 1. As this was an exploratory study no power calculation could be performed to determine the number of participants needed. We aimed to ensure that we recruited six patients to each group. Given an assumed VAP rate of 25% this meant that we needed to recruit at least 24 patients in the brain injured group.

### 2.2 Nuclear Magnetic Resonance Spectroscopy

Full details of the proton Nuclear Magnetic Resonance Spectroscopy (^1^H NMR) processing can be found in the Supplemental material. Samples were prepared following the new protocols designed for Phenome Centres and Invitro Dagnostic research (IVDr) as previously described (Dona et al., 2014). Prior to analysis samples were randomized and blinded to the patient groupings. All ^1^H NMR experiments were performed using a Bruker Avance III 600 spectrometer working at 14.1 T equipped with a BBI probe. Three different ^1^H NMR spectra of serum were obtained for each sample with a total acquisition time of 12 min. These three experiments were i) a general profile of the human serum obtained with a 1D pulse sequence using the first part of a Nuclear Overhauser Effect pulse sequence to include two different water presaturation periods and improve signal cancellation; ii) a spin-echo experiment using the relaxation edited Carr-Purcell-Meiboom-Gill (CPMG) pulse sequence, allowing low molecular weight species to be detected by eliminating fast relaxing signals arising from large molecules such as proteins; and iii) a 2D *J* resolve experiment which helps with structure elucidation. ^1^H NMR is a robust and reliable technique that is very reproducible, however, biofluid samples may be subject to chemical change over time once thawed (Dona et al., 2014) so the samples were kept at 5 °C immediately prior to analysis to limit this. For all spectra the region from 0·1-10 ppm, to exclude the peak due to TSP, was divided into approximately 40,000 data points. The water signal region, 4·5–4·85 ppm, was removed prior to further processing. All samples underwent probabilistic quotient (median fold) normalization (Dieterle et al., 2006).

To aid metabolite identification further 2D experiments were carried out on selected samples. ^1^H—^1^H Correlation Spectroscopy (COSY), to demonstrate proton spins that are directly coupled to each other, and Total Correlation Spectroscopy (TOCSY), to demonstrate coupled protons up to 6 bonds apart within a molecule, experiments were performed.

### 2.3 Statistical Analysis

In multivariate models, analytes with naturally higher concentrations tend to be associated with higher variance. By standardizing the variance those variables with generally lower values can be given similar weight to those variables with higher concentrations. Within the model this was achieved by scaling spectral data to unit variance. Initial exploration of the data with principal component analysis (PCA) was performed to look for natural clustering and to detect outliers before supervised multivariate analysis using orthogonal partial least squared discriminant analysis (OPLS-DA). Outliers were each inspected to look for reasons that they may be different from the rest of the data set. The data were inspected manually to look for errors in processing. Outliers were only excluded if there was a technical reason that their data was inaccurate. OPLS-DA generated models to optimally detect the metabolites associated with differentiation between the predefined groups; pneumonia, brain injuries and those developing VAP. OPLS-DA models were cross validated using seven fold cross-validation using a “leave-one-out” methodology. To assess the reliability of the models a cross-validated analysis of variance was used (CV-ANOVA) which analyses whether the model has significantly smaller cross validated predictive residuals than the variation around the global average, as would be expected if the model was generated by chance (Umetrics, 2013). Permutation testing was also performed where the Y variables were randomly generated 20 times, in order to scramble the true class information, and a new model constructed for each permutation. The *Q*^2^ and *R*^2^ could then be compared with those generated from the random models. To determine the discriminant potential of the OPLS-DA models Receiver Operating Characteristic (ROC) analysis was performed on the cross validated data.

Important spectral signals were identified by examining the loadings associated with each model and the most discriminatory metabolites were selected by picking those associated with the highest correlation coefficients. All multivariate analysis was performed using the SIMCA 13·0 statistical package (Umetrics, Sweden). Univariate comparison of metabolites was performed on full profile NMR data (~ 4 K data points). These were compared using analysis of variance (ANOVA) with false discovery rate (FDR) correction using previously described methods (Storey, 2002) using Matlab®, its statistical package and in-house scripts.

For those metabolites appearing to discriminate between groups the integrals of the relevant spectral regions were calculated. These allowed relative concentration to be calculated for further univariate analysis. These comparisons were made using Student's *t*-test and Bonferroni's correction was applied to account for multiple comparisons.

### 2.4 Metabolite Identification

Important signals were identified using a combination of techniques. Initially Statistical Total Correlation Spectroscopy (STOCSY) using an in-house script, MatLab 2013 (MathWorks, Massachusetts, USA) was used to look for peak correlation within the acquired spectra (Cloarec et al., 2005). The statistical correlations were validated experimentally using 2D NMR spectra (a set of COSY, TOCSY and ^1^H,^13^C–HSQC experiments were performed on selected samples) Metabolites were then found in the SBASE database using AMIX 3·9·11 software (Bruker BioSpin, Germany) or in published literature (Nicholson et al., 1995, Macintyre et al., 2010).

### 2.5 Lipoprotein Analysis

Lipoprotein subclass analysis was performed using the 1D general profiling of serum samples and the *B.I.-LISA* (Bruker IVDr Lipoprotein Subclass Analysis) platform. B.I.-LISA uses a PLS-2 regression model to fit the area under the curve of the —CH_3_ and —CH_2_— signals of lipoproteins appearing around 0.87 and 1.25 ppm, respectively. This regression model was trained using the ^1^H NMR spectra obtained for the 105 lipoprotein subfractions obtained via ultracentrifugation for 200 different original plasma samples (Bruker, 2016). The spectra of the lipoprotein ultracentrifugation fractions were studied together with the ^1^H 1D general profile of each sample. The 105 different subclasses are partially correlated and they should not be considered as completely independent analytes because of the nature of the lipoprotein subclasses obtained by ultracentrifugation and due to the PLS-2 regression model used for the correlation between spectra and lipoprotein subclass identification. Quantitative values were obtained for 105 fractions including triglycerides, cholesterol, low density lipoproteins (LDL), very low density lipoproteins (VLDL), intermediate density lipoproteins, and high density lipoproteins (HDL). Statistical comparison was made using a combination of multivariate analysis, PCA and OPLS-DA using SIMCA 13.0 (Umetrics, Data Analytics, Sweden), and univariate analysis. ANOVA with correction for false discovery rate, using the Benjamini Hochberg method, was carried out on the 105 lipid fractions using Matlab®, its statistical package and in-house scripts and R version 2.15.2 (The R Foundation). Univariate analysis of broad classes of lipoproteins was performed using Student's *t*-test with Bonferroni's correction for multiple comparisons using Excel 2010 (Microsoft, USA).

## 3 Results

### 3.1 Patients

Of the 45 patients recruited, 15 were assigned to the pneumonia group and 26 had brain injuries with no evidence of infection at admission. One patient withdrew consent and three patients did not have sufficient serum to allow NMR analysis. Of the brain injured patients, seven subsequently developed VAP a mean of 127 h after the start of ventilation. Patients were similar across the groups with respect to their demographic details, Table 1. Features that differed between the pneumonia and VAP groups from those with brain injuries included markers of infection, as would be expected. In all of the comparisons below no technical inaccuracies were found to account for any outliers so all cases have been included in the analyses.

### 3.2 Comparison of Pneumonia with Brain Injury

**Fig. 2:**
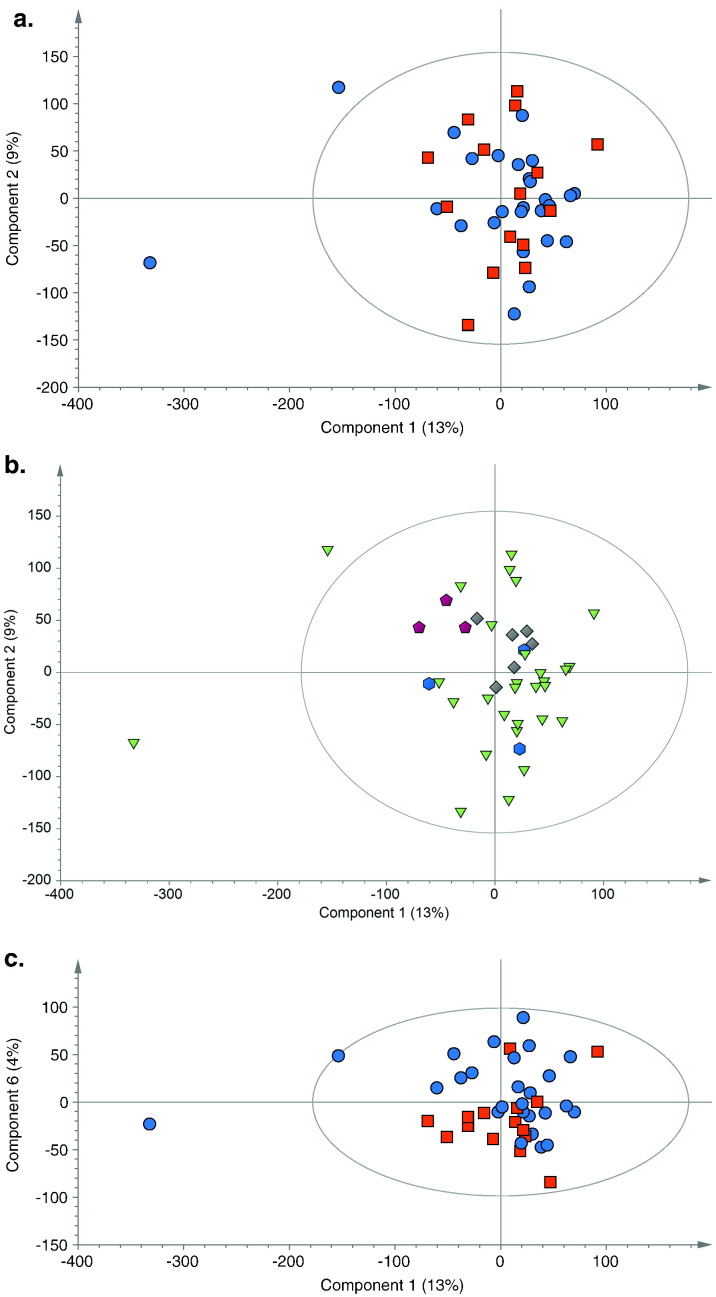
PCA scores plot (R^2^X = 0·42 Q^2^ = 0·06) showing a. first and second principal components, comparing samples taken at the first time point from patients admitted with brain injuries (blue circles) to those with pneumonia (red squares). b. PCA scores plot colored according to ethnicity, White (green triangles), Afrocaribbean (grey diamonds), Indian Asia (purple pentagons) and others (blue hexagons) c. First and sixth components of the PCA scores plot for samples taken at the first time point from patients admitted with brain injuries (blue circles) and those with pneumonia (red squares). Dots representing samples taken from patients with pneumonia have a tendency to cluster in the lower half of the plot.

**Fig. 3:**
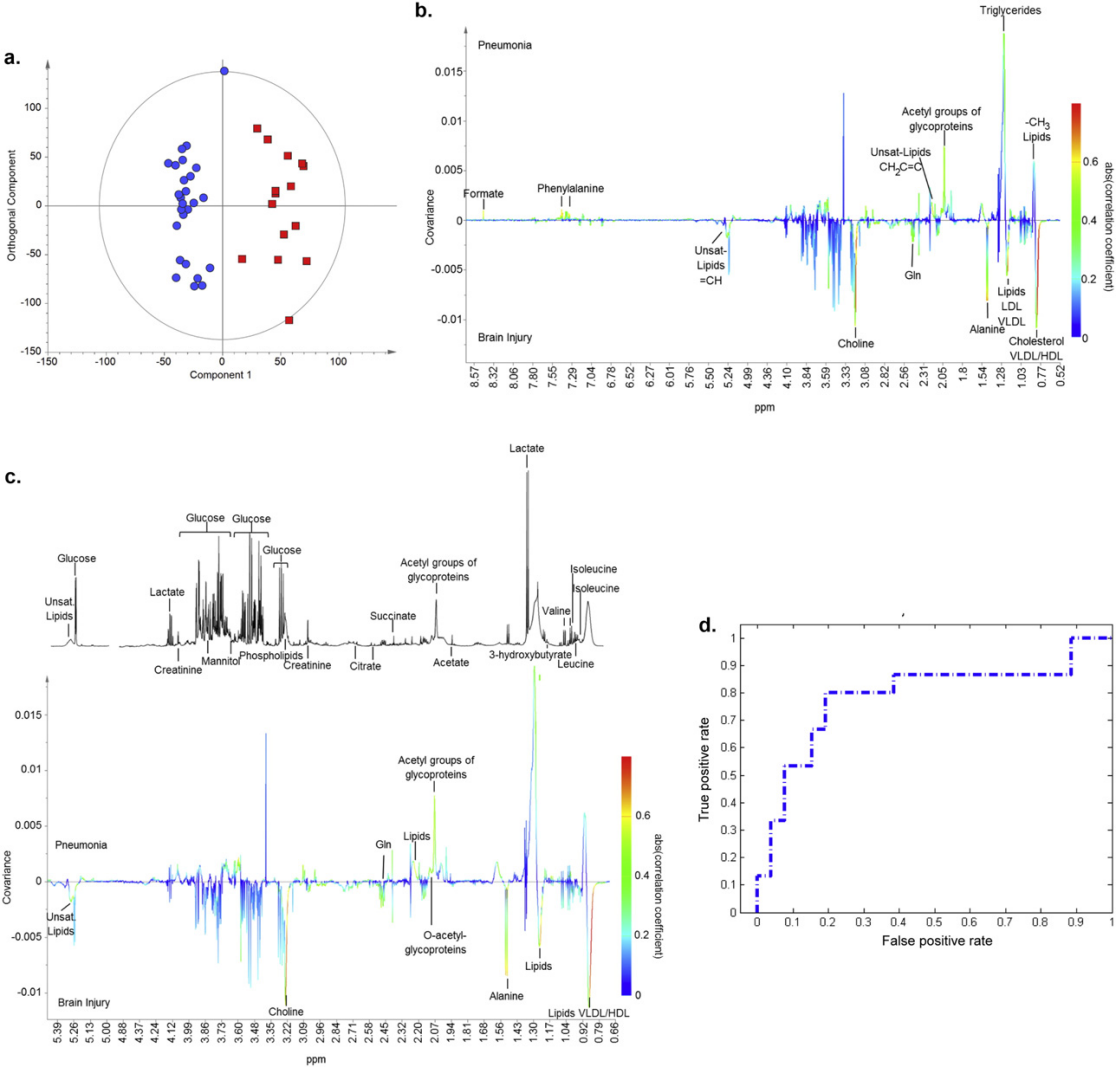
a) OPLS-DA scores plot, with one orthogonal and one aligned component (R^2^Y = 0·91, Q^2^Y = 0·28, *p* = 0·02) comparing samples taken at the first time point from patients admitted with brain injuries (blue circles) to those with pneumonia (red squares). b) Regression coefficient plot colored according to the correlation between the metabolic NMR data and the class information. Metabolites present in relatively higher concentrations in the pneumonia group deflect upwards and in brain injuries downwards. The strength of the correlation of metabolites to this model is given by the intensity of the colour of the peak with red representing the strongest correlation and dark blue no correlation. c) Magnified portion of the regression coefficient plot between 0.65 and 5.40 ppm with reference spectra. d) ROC curve for the cross validated scores from 3a (AUC 0.78).

Metabolic profiles of patients with pneumonia were compared to those from patients with brain injuries using PCA (*R*^2^*X* = 0.42, *Q*^2^ = 0.06). It demonstrated a degree of natural separation when the first and sixth components were compared, Fig. 2. When the first component was examined some clustering may be explained by ethnic differences in the patient population with Indian Asian and Afrocaribean patients independently clustering. The low *Q*^2^ value is indicative that this group of patients are metabolically highly variable. OPLS-DA modelling of the two groups (*R*^2^*Y* = 0.91, *Q*^2^*Y* = 0.28, *p* = 0.02), showed some discriminant capacity with an area under the ROC curve of 0.78, Fig. 3a,d. This model was improved when only cases that could be classified with CPIS were used (*R*^2^*Y* = 0.93, *Q*^2^*Y* = 0.43, *p* = 0.01). The loadings from these models indicated that different species of lipids predominated in the two groups and phospholipids, glutamine and alanine predominated in those with brain injuries, and formate, glycoproteins and phenylalanine in those with pneumonia (Fig. 3b and c). Since the spectra were acquired using a CPMG pulse sequence, designed to minimize the contribution form macromolecular species such as lipids, the lipid signature observed indicates that the discriminatory lipids were smaller, more mobile species. Within Fig. 3a one of the brain injured patients sits between the two groups, this patient was one of the brain injured patients who developed VAP and it is possible that at enrolment they had subclinical features of pneumonia. When the model was reconstructed without this sample the predictive capacity improved but the metabolites of importance remained the same (*R*^2^*Y* = 0.94, *Q*^2^*Y* = 0.34, *p* < 0.01).

Univariate comparison of full NMR data using a false discovery rate cut off of 5% confirmed the differences in metabolites identified previously with lipid species, phospholipids (choline), alanine and phenylalanine remaining significantly different between those admitted with pneumonia and brain injuries, Table 1 in Supplementary material. Comparison of relative concentrations of discriminant metabolites showed a trend towards glycoproteins being at higher concentration in those with pneumonia and glutamine being at higher concentration in those with brain injuries, Table 2, however, none of these comparisons retained statistical significance after Bonferroni's correction was applied, with a target *p*-value of 0.005.

### 3.3 Lipoprotein Analysis

As lipid species appeared to be discriminating metabolites, detailed lipid analysis was performed as described above. Comparison of total plasma levels of lipids, Table 3, showed a trend towards elevated levels of triglycerides and reduced total, LDL and HDL cholesterol in those with pneumonia compared to the brain injured patients. However, none of these changes reached statistical significance when a *p*-value threshold of 0.0125 was used, taking into account Bonferroni's correction for multiple comparisons. When OPLS-DA (*R*^2^*Y* = 0.31, *Q*^2^*Y* = 0.19, *p* = 0.02) was used to analyze 105 lipid components, some of the most discriminant lipids could be seen to be free and total cholesterol, apolipoprotein A1, and phospholipids within HDL-4, free cholesterol in HDL-3 and LDL-6, and total and HDL-4 apolipoprotein A2 within the brain injured group. Triglycerides within LDL-2, and HDL-1 and free cholesterol in VLDL-5 were discriminant in the pneumonia group, Fig. 2 in Supplementary appendix. These findings were confirmed with ANOVA with a false discovery rate of 5%. In total 27 of the lipid species showed statistically significant differences between pneumonia and brain injured patients with this analysis, Table 3 below and Fig. 2 in Supplementary appendix.

### 3.4 Comparison of Brain Injury with VAP

**Fig. 4:**
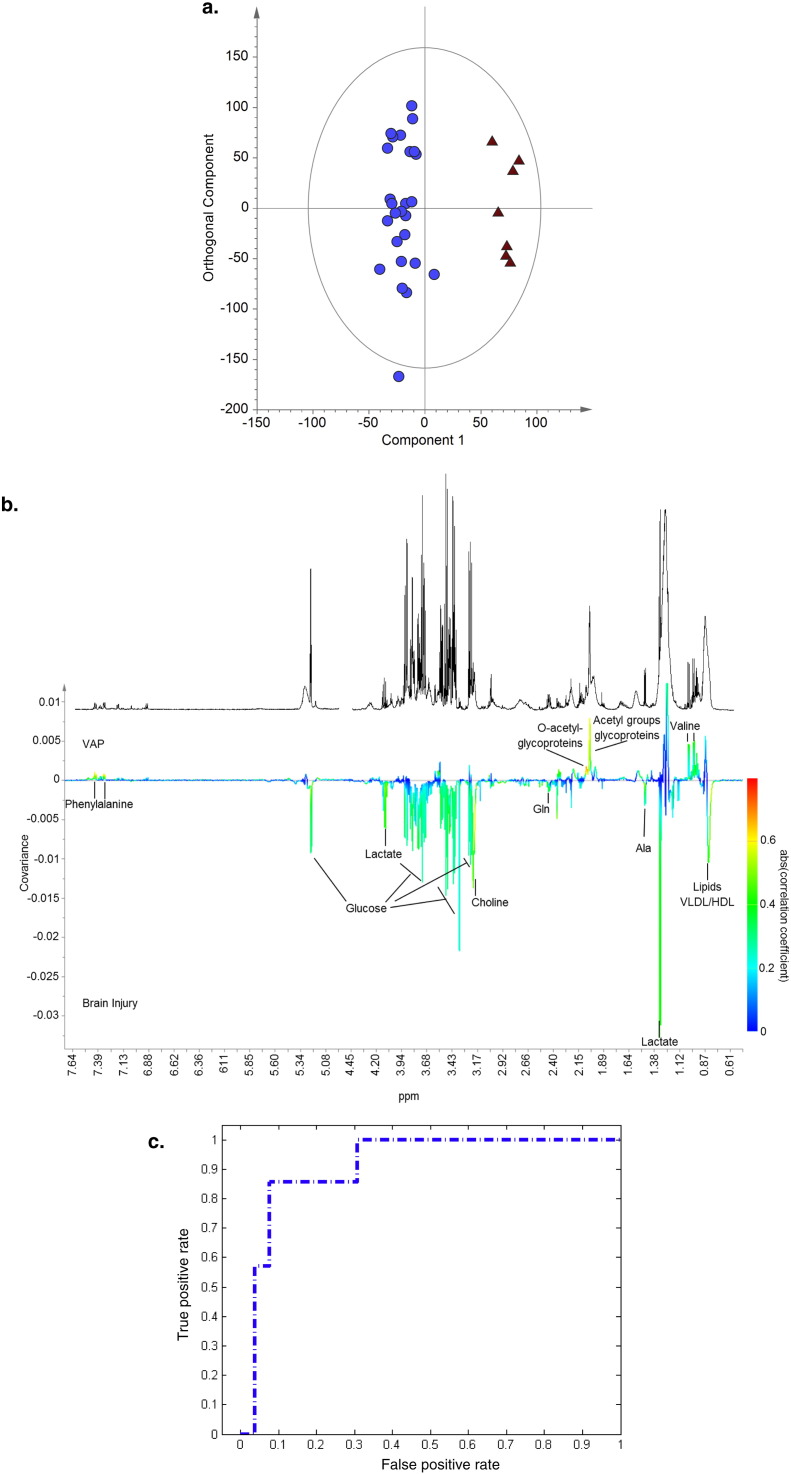
a) OPLS-DA scores plot with one orthogonal and one aligned component, (R^2^Y = 0·94, Q^2^Y = 0·27, *p* = 0·05) comparing samples taken at the first time point from patients admitted with brain injuries (blue circles) to those with VAP (red triangles). b) Regression coefficient plot, with reference spectrum, colored according to the correlation between the metabolic NMR data and the class information. Metabolites dominating in the VAP group deflect upwards and in brain injuries downwards. The strength of the correlation of metabolites to this model is given by the intensity of the colour of the peak with red representing the strongest correlation and dark blue no correlation. c) ROC curve for the cross validated scores from 4a (AUC 0.91).

Samples taken at the time VAP developed from the seven patients who developed VAP were compared to those from all brain injured patients at the start of ventilation. An OPLS-DA model could be constructed (*R*^2^*Y* = 0.94, *Q*^2^*Y* = 0.27, *p* = 0.05), Fig. 4a, with ability to separate the two groups with an area under the ROC curve of 0.91, Fig. 4c. The *Q*^2^ improved if only brain injured patients who did not develop VAP or other infection were used as the control group (*R*^2^*Y* = 0.96, *Q*^2^*Y* = 0.38, *p* = 0.07). Including patients classified only using CPIS failed to improve either of these models. Although none of the metabolites in the VAP and brain injury model had such strong discriminating potential as those when pneumonia was compared to brain injuries, the important differentiating metabolites were similar. Lipids, choline, and lactate predominated in those with brain injuries and glycoproteins, phenylalanine, and valine were more abundant in those with pneumonia (Fig. 4b). However, when univariate comparisons of whole spectral data were made none of the metabolites reached statistical significance after correction for false discovery rate, Table 2 in Supplementary material, suggesting this model may not be as robust as that comparing pneumonia to brain injuries. When comparison was made of the relative concentrations of the most discriminant metabolites from the OPLS-DA models valine and N- and O-glycoproteins all were significantly more abundant in those with VAP than those with brain injuries at a *p*-value of 0.0036 after Bonferroni's correction, Table 4.

### 3.5 Lipoprotein Analysis

Comparison of total plasma levels of lipids showed a trend towards elevated levels of triglycerides and reduced total, LDL, and HDL cholesterol in those with VAP, Table 5. Only the changes in HDL cholesterol reached statistical significance (*p* < 0.001) after Bonferroni's correction. When samples from patients with brain injuries, who did not develop infection, were used that reflected a similar length of ICU stay as those samples from patients with VAP, similar trends were seen with reduction in total, HDL, and LDL cholesterol and increased triglycerides in those with VAP. In brain injured patients without infection triglycerides, total, and LDL cholesterol levels increased and HDL cholesterol levels decreased with time in ICU.

When OPLS-DA (*R*^2^*Y* = 0.63, *Q*^2^*Y* = 0.47, *p* < 0.01) was used to analyze 105 lipids, the most discriminant lipids were VLDL-4 and 5 free and total cholesterol, triglycerides within HDL-2, HDL-3, LDL-1 and HDL and intermediate density lipoprotein total and free cholesterol, apolipoprotein-B, and phospholipid in those with VAP and HDL-4 total cholesterol, and HDL and HDL-4 free cholesterol in those with brain injuries. These findings were confirmed with ANOVA with a false discovery rate of 5%. In total 34 of the lipid species showed statistically significant differences between VAP and brain injured patients with this analysis, Table 5 below and Fig. 4 in Supplementary appendix.

### 3.6 Comparison of Pneumonia with VAP

When samples taken from patients with VAP were compared to those taken from patients admitted with pneumonia it was impossible to build an OPLS-DA model to separate the two groups, either with the whole metabolic data or with the lipid data, suggesting that the metabolites that are important in both conditions are similar. A larger cohort of patients would be necessary to observe difference in the metabolic profiles of these two groups as they are more subtle. It is encouraging, however, that we observed some differences in the small molecule profiles of the two groups when each was compared to brain injuries with both multivariate and univariate analysis.

## 4 Discussion

This study examined the ability of ^1^H NMR spectroscopy of serum from patients in intensive care to differentiate those with and without pneumonia and specifically to determine those developing VAP amongst a group of patients with brain injuries. Previously only a limited amount of work focusing on metabolic profiling of biofluids from critically unwell patients with sepsis (Mickiewicz et al., 2013, Mickiewicz et al., 2014, Blaise et al., 2013, Neugebauer et al., 2016, Seymour et al., 2013) has been done and with little focusing on pneumonia and nothing specifically investigating VAP.

Multivariate and univariate analysis of serum metabolic profiles showed ability to separate pneumonia from brain injuries at the time of admission, this discrimination was supported by the natural separation seen on PCA. However, models looking at VAP were less robust with no differences being found on univariate comparison of whole spectral data. The metabolites that appeared important in both sets of multivariate models were similar. Lipid species were important in both comparisons and phenylalanine and glycoproteins appeared more abundant in both pneumonia and VAP compared to brain injury. Phospholipids - especially choline, alanine and glutamine were all more predominant in those with brain injuries compared to either pneumonia or VAP. Although formate appeared to aid in the separation of pneumonia from brain injury, at admission, this metabolite did not appear important in comparisons with VAP and similarly valine appeared important in those with VAP but not those admitted with pneumonia.

Several of the metabolites causing separation were lipid species. Patients with pneumonia have been seen to have lower total HDL and LDL cholesterol and higher triglyceride concentrations than those without (Gruber et al., 2009, Deniz et al., 2006), findings supported by results from this study. Circulating lipids provide a source of energy and may also have a role in regulating the immune response, such as by binding endotoxin (Dong et al., 2012).

Glycoproteins have an important role in inflammation, and their synthesis and secretion increases during inflammatory processes (Connelly et al., 2016). We observed that the levels of circulating glycoproteins were higher in patients with pneumonia than in those without. Patients who developed VAP had both elevated levels of *N*-acetyl glycoproteins and also *O*-acetyl glycoproteins which are normally intracellular and within the extracellular matrix. Glycoproteins are potential biomarkers for inflammation (Otvos et al., 2015) and our results suggest that signals belonging to *N*-acetyl and *O*-acetyl groups from glycoproteins could be use as markers for pneumonia and in particular VAP.

Other metabolites that discriminated patients with pneumonia and VAP were phospholipids, especially choline, and amino acids, in particular phenylalanine, alanine, and glutamine. Phenylalanine is elevated in sepsis (Mickiewicz et al., 2013, Mickiewicz et al., 2014), possibly representing a reduction in its conversion to tyrosine, an increase in oxidative stress (Neurauter et al., 2008), as a result of immune activation (Ploder et al., 2008), or due to alterations in the catabolism of skeletal muscle. Alanine and glutamine were reduced in those with pneumonia as in previous sepsis studies (Mickiewicz et al., 2013, Mickiewicz et al., 2014). These changes may reflect alterations in nutritional status in these conditions and the alteration in the release of amino acids from muscle proteins. Our data also suggested phospholipids may be reduced in those with pneumonia compared with those with brain injury, a consistent finding with work differentiating pneumonia from other causes of sepsis (Neugebauer et al., 2016).

Comparison of these data to previous studies demonstrates a number of differences. Analysis of plasma from children with pneumonia (Laiakis et al., 2010) found elevated uric acid, hypoxanthine and glutamic acid levels with decreases in adenosine diphosphate and tryptophan. Adults in ICU (Seymour et al., 2013) with community acquired pneumonia had higher levels of bile acids, metabolites of steroid metabolism and metabolites related to oxidative stress in non-survivors. Differences in these findings may be because the previous studies used different control groups to the current study and because mass spectrometry was used for analysis. Mass spectrometry is able to detect metabolites at lower concentrations than NMR. However, some metabolites are unsuitable for analysis with mass spectrometry due to their non-volatility (GC-MS), their ability to be ionized, or they can be influenced by ion suppression. Moreover, NMR allows direct quantification of metabolites whereas MS requires targeted assays to achieve quantification. Future work would ideally use parallel sample profiling using both techniques. Other differences in the metabolic markers of pneumonia may arise from the different patient populations, or differences due to comparing survivors to non-survivors as opposed to infected versus non-infected. Differences in causative organisms between studies may result in different metabolites being important, as organisms can be differentiated based on metabolic profiling (Slupsky et al., 2009a). This is especially relevant when considering the ability of metabolic profiling to differentiate community acquired and ventilator acquired pneumonias. Community acquired pneumonia is mostly caused by *Pneumococcus* and *Haemophilus* whereas VAP is often caused by gram negative organisms, and resistant organisms such as *Staphylococcus aureus*. Microbiological results from this study reflected this heterogeneity. The metabolic phenotype of pneumonia may vary depending on the microorganism causing the infection. A larger study will be necessary to stratify the patients with pneumonia according to the organisms causing the disease and define metabolic phenotypes associated with each.

Many of the metabolic changes were similar in the models differentiating critically ill patients with VAP from those without. Although the discriminant capacity of these models was limited, they were based on a small sample of patients and represent the first attempt at using these methods in this condition.

Limitations of this study must be considered. The small number of patients in each group, especially those with VAP, where there were just seven patinets, could have led to biased model estimates, this may be represented by the differences seen between the *R*^2^ and *Q*^2^ values in these models. Because of the limited number of patients in each group we were unable to provide a validation cohort which would have been the ideal method to test the models. However, the current data are a promising starting point from which to perform a larger study. In this study NMR was the analytical platform of choice. Although NMR is both reliable and reproducible it lacks the sensitivity of mass spectrometry (MS) and is not an ideal platform to identify specific lipids, apart from the lipoprotein subclasses. Further work would employ parallel analysis with both NMR and MS and the application of Liquid Chromatography – Mass Spectrometry lipidomic analysis to specifically investigate the role of lipid species in more detail. The study used a group of brain injured patients as a control group and it must be recognized that the findings may represent changes seen in brain injury as opposed to changes in pneumonia. However, the fact that similar changes were seen in those with VAP, all of whom also had a brain injury, to those with pneumonia lends some support to the findings being related to the pneumonic process. Further work looking at different cohorts of intensive care patients needs to be done to clarify the specificity of our findings to pneumonia. Finally as no true “gold standard” tests exist by which to diagnose VAP or pneumonia, the comparison groups in this study may not be entirely discrete. However, every effort was made to ensure correct classification of patients using a combination of clinical “bedside” opinion, objective CPIS and independent assessment. This work looked at VAP in a cohort of brain injured patients, further work will be needed to establish if the current findings apply to other groups of critically ill patients and to all populations. Some clustering was seen in our data, potentially accounted for by ethnicity, which may mean a larger study is needed to accurately stratify patients based on such demographic details.

## 5 Conclusion

^1^H NMR analysis of serum from critically ill patients showed an ability to differentiate those with pneumonia from those without. These techniques may also be beneficial in determining those patients who go on to develop VAP and NMR spectroscopy has the potential to become a useful diagnostic tool in critical care.

## Contributors

DA, BJ and AG designed the study. DA recruited all the patients and collected all clinical samples. DA and BJ analyzed the data, KV wrote statistical programs used in the analysis of the data and performed some of the analysis. DA, BJ, EH, AG interpreted the data analysis. DA wrote the first draft of the manuscript. All authors reviewed the manuscript for important intellectual content and approved the final version for publication.

## Declaration of Interests

We declare that we have no competing interests.

## Figures and Tables

**Table 1:** Clinical features of included patients. Continuous variables are given as mean and standard deviation and categorical variables as number and percentage. *p*-Values presented in bold, italic text relate to parameters that were significant at the *p* < 0.05 level.

	Pneumonia (P)	All brain injury (BI)	*p*-Value (BI vs P)	Brain injury excluding those developing VAP (exBI)	VAP	*p*-Value (exBI vs VAP)
N	15	26	–	19	7	–
Age (mean ± SD)	54.1 ± 15·6	55.0 ± 15.8	0.86	56.3 ± 16.4	51.7 ± 14·9	0.51
Sex, number of males (%)	11 (73.3)	15 (57.7)	0.50	11 (57.9)	4 (57.1)	1.00
Ethnicity, number White European (%)	10 (66.7)	18 (69.2)	1.00	12 (63.2)	6 (85.7)	0.37
APACHIE II Score (Mean ± SD)	19.8 ± 5.8	17.5 ± 5.5	0.21	17.9 ± 4.2	16.3 ± 8.5	0.64
SOFA Score (Mean ± SD)	10.5 ± 3.1	8·8 ± 2.7	0.09	8.5 ± 2.6	8.6 ± 2.6	0.93
CPIS (Mean ± SD)	5.7 ± 1.0	2.5 ± 1.6	**<* 0***.***001***	2.1 ± 1.5	6.3 ± 1.8	**<* 0***.***001***
Lowest WCC (10^9^/L) (Mean ± SD)	14.5 ± 7.5	10.6 ± 4.6	0.08	10.3 ± 4.2	11.2 ± 3.1	0.58
Highest WCC (10^9^/L) (Mean ± SD)	15.3 ± 7.1	11.7 ± 4.8	0.09	11.1 ± 4.3	11.2 ± 3.1	0.96
Lowest CRP (mg/L) (Mean ± SD)	147.6 ± 108.0	46.9 ± 49.5	***0***.***003***	40.4 ± 36.3	133.2 ± 87.2	***0***.***03***
Highest CRP (mg/L) (Mean ± SD)	175.5 ± 92.5	59.6 ± 48.8	***< 0***.***001***	51.4 ± 40.4	133.2 ± 87.2	***0.05***
Lowest temperature (°C) (Mean ± SD)	36.0 ± 0.9	36.0 ± 0.7	0.91	36.0 ± 0.6	36.1 ± 1.3	0.85
High temperature (°C) (Mean ± SD)	37.9 ± 1.0	37.3 ± 0.9	0.07	37.2 ± 1.0	38.2 ± 1.1	0.08
Lowest PaO2:FiO2 (kPa) (Mean ± SD)	24.0 ± 8.9	40.4 ± 14.6	***< 0***.***001***	43.0 ± 12.2	23.4 ± 11.3	***0***.***003***
Lowest MAP (mmHg) (Mean ± SD)	71.6 ± 9.7	74.2 ± 10.8	0.43	75.7 ± 10.8	71.4 ± 12.5	0.44
Use of noradrenaline, N (%)	11 (73.3)	16 (61.5)	0.51	12 (63.2)	3 (42.9)	0.41
Use of antibiotics N (%)	15 (100.0)	11 (42.3)	***< 0***.***001***	8 (42.1)	7 (100.0)	***0***.***01***
Enteral nutrition, N (%)	13 (86.7)	19 (73.1)	0.45	15 (79.0)	7 (100.0)	0.55
Time to sampling from start of ventilation (h) (Mean ± SD)	44.3 ± 9.2	41.9 ± 16.6	0.55	43.8 ± 16.9	138.3 ± 43.2	***< 0***.***001***
Time of day of sample, number taken in the morning (%)	9 (60.0)	16 (61.5)	1.00	12 (63.2)	7 (100.0)	0.13

**Table 2:** Univariate comparision of spectral integrals for discriminant metabolites on OPLS-DA comparing pneumonia to brain injury. Data are given as relative concentration with regard to patinets with pneumonia.

Metabolite	ppm	Relative concentration in pneumonia	*p*-Values
Alanine	1.456–1.489	− 1.17	0.06
N-Glycoproteins	2.022–2.054	1.18	0.02
O-Glycoproteins	2.058–2.079	1.14	0.04
Glutamine	2.426–2.468	− 1.26	0.04
Choline	3.018–3.213	− 1.10	0.15
P-Choline	3.213–3.224	− 1.10	0.74
Unidentified metabolite	3.570–3.575	1.29	0.03
Phenylalanine	7.311–7.338	1.23	0.22
Phenylalanine	7.348–7.385	1.12	0.36
Formic Acid	8.451–8.456	2.07	0.18

**Table 3:** a. Total levels of lipids in those with brain injuries and those with pneumonia. b. Lipoprotein subfractions whch were most discriminant in the OPLS-DA model with the relative concentrations in pneumonia compared to brain injury. P- and q-values are given for ANOVA with correction for false discovery rate. Concentrations given as mean ± standard deviation.

a			
	Brain injury	Pneumonia	*p*-Value
N	26	15	–
Total triglycerides (mg/dl)	152.0 ± 71.0	181.7 ± 90.9	0.29
Total cholesterol (mg/dl)	215.2 ± 75.1	167.4 ± 54.2	0.02
LDL cholesterol (mg/dL)	111.0 ± 65.1	70.0 ± 44.2	0.02
HDL cholesterol (mg/dL)	78.6 ± 24.5	54.4 ± 29.9	0.013


b				
Lipid species	Brain injury	Pneumonia	*p*-Value	q-Value
N	26	15	–	–
H4FC in mg/dL	5.5 ± 2.1	2.3 ± 2.2	4.2 × 10^− 5^	0.003
H4A1 in mg/dL	84.8 ± 24.1	46.8 ± 29.3	0.0001	0.003
H4CH in mg/dL	21.7 ± 7.9	9.8 ± 10.4	0.0002	0.006
H4PL in mg/dL	30.1 ± 9.8	16.9 ± 11.4	0.0004	0.009
V5FC in mg/dL	0.5 ± 0.3	1.0 ± 0.5	0.0009	0.020
H4A2 in mg/dL	21.8 ± 6.9	12.9 ± 9.4	0.0013	0.020
H3FC in mg/dL	3.9 ± 1.3	2.4 ± 1.4	0.0014	0.020
L2TG in mg/dL	2.9 ± 1.9	6.1 ± 4.4	0.0024	0.031
L6FC in mg/dL	9.2 ± 5.4	4.1 ± 4.0	0.0031	0.033
TPA2 in mg/dL	45.6 ± 8.1	35.8 ± 11.9	0.0032	0.033
H1TG in mg/dL	6.1 ± 2.8	9.8 ± 5.0	0.0036	0.033

**Table 4:** Univariate comparision of spectral integrals for discriminant metabolites on OPLS-DA analysis comparing VAP to brain injury. Data are given as relative concentration with regard to patinets with VAP.

Metabolite	ppm	Relative concentration in VAP	*p*-Values
Valine	0.976–0.994	1.44	< 0.001
Vaine	1.020–1.045	1.45	< 0.001
Alanine	1.456–1.489	1.01	0.88
N-Glycoproteins	2.022–2.054	1.36	< 0.001
O-Glycoproteins	2.058–2.079	1.36	< 0.001
Glutamine	2.426–2.468	− 1.08	0.71
Choline	3.018–3.213	− 1.03	0.69
P-Choline	3.213–3.224	− 1.33	0.04
Unidentified metabolite	3.570–3.575	1.30	0.003
Lactate	4.084–4.111	− 1.13	0.36
Glucose	5.220–5.340	1.19	0.52
Phenylalanine	7.311–7.338	1.12	0.58
Phenylalanine	7.348–7.385	1.26	0.14
Phenylalalnine	7.403–7.444	1.09	0.45

**Table 5:** a. Total levels of lipids in those with brain injuries and those with VAP. b. Lipoprotein subfractions whch were most discriminant in the OPLS-DA model with the relative concentrations in VAP compared to brain injury. P- and q-values are given for ANOVA with correction for false discovery rate. Concentrations given as mean ± standard deviation.

a			
	Brain Injury	VAP	*p*-value
N	26	7	–
Total triglycerides (mg/dL)	152.0 ± 71.0	198.6 ± 53.3	0.08
Total cholesterol (mg/dL)	215.2 ± 75.1	185.3 ± 45.1	0.20
LDL cholesterol (mg/dL)	111.0 ± 65.1	77.6 ± 29.2	0.06
HDL cholesterol (mg/dL)	78.6 ± 24.5	46.1 ± 15.3	< 0.001


b				
Lipid species	Brain injury	VAP	*p*-Value	q-Value
N	26	7	–	–
IDCH in mg/dL	9.7 ± 7.2	26.9 ± 6.5	3.1 × 10^− 6^	0.0003
IDAB in mg/dL	3.5 ± 2.5	8.7 ± 2.0	1.6 × 10^− 5^	0.001
IDPL in mg/dL	6.3 ± 4.2	14.8 ± 4.2	4.4 × 10^− 5^	0.002
H2TG in mg/dL	3.0 ± 1.3	5.3 ± 1.2	0.0002	0.005
H3TG in mg/dL	2.7 ± 1.3	5.0 ± 1.3	0.0002	0.005
IDFC in mg/dL	2.1 ± 2.2	5.8 ± 2.0	0.0004	0.007
V5CH in mg/dL	0.9 ± 1.0	3.0 ± 2.0	0.0006	0.007
L1TG in mg/dL	6.0 ± 4.4	13.5 ± 5.5	0.0006	0.007
V4FC in mg/dL	2.3 ± 2.0	5.8 ± 2.8	0.0007	0.007
V5TG in mg/dL	3.2 ± 1.6	5.9 ± 1.9	0.0008	0.007
H4CH in mg/dL	21.7 ± 7.9	9.2 ± 7.7	0.0008	0.007
H4FC in mg/dL	5.5 ± 2.1	2.3 ± 2.0	0.0010	0.009
HDTG in mg/dL	14.9 ± 6.1	23.9 ± 5.1	0.0012	0.009
V4CH in mg/dL	4.8 ± 4.1	11.7 ± 6.0	0.0013	0.009
HDFC in mg/dL	20.8 ± 7.4	10.5 ± 4.9	0.0016	0.011

## Appendix A Supplementary data

Supplementary material Image 1
