# Supplementary material for: Metabolic Profiling in Patients with Pneumonia on Intensive Care
Source: eBioMedicine. 2017 Mar 29;18:244–53. doi: 10.1016/j.ebiom.2017.03.034 (PMC5405179; doi:10.1016/j.ebiom.2017.03.034)
Supplement: Supplementary file 1 — Supplementary material [file mmc1.docx]

**Supplementary Material**

**Details of NMR analysis**

A volume of 300 µL of serum was mixed with 300 µL of H_2_O:D_2_O, in a 90:20 ratio, buffer containing 75 mM of NaH_2_PO_4_, 3-(trimethyl-silyl) propionic acid (TSP) and NaN_3_ at pH 7.4 ([Dona et al., 2014](#_ENREF_3)). Samples were vortexed and centrifuged at 12000 g for five minutes to remove solid material. An aliquot of 550 µL of the supernatant was placed into 5 mm outer diameter NMR tubes and immediately loaded onto a refrigerated SampleJet robot (Bruker Corporation, Germany) and kept at 5°C until measurement. All measurements were carried out within 24h of sample preparation. All ^1^H-NMR experiments were performed using a Bruker Avance III 600 spectrometer working at 14.1 T equipped with a BBI probe. ^1^H-NMR spectra of serum samples were collected at a constant temperature of 310K using a 1D pulse sequence using the first part of a Nuclear Overhauser Effect pulse sequence to achieve presaturation of the water peak and obtain the general profile of the human blood serum sample, a relaxation edited Carr-Purcell-Meiboom-Gill (CPMG) pulse sequence, allowing low molecular weight species to be detected by eliminating fast relaxing signals arising from large molecules such as proteins, and a 2D *J* resolve experiment which helps with structure elucidation.([Beckonert et al., 2007](#_ENREF_1), [Dona et al., 2014](#_ENREF_3)). A total of 32 free induction decays (FID) were acquired for each experiment into 96 K data points using a 20 ppm spectral width centred at 4.75 ppm. The relaxation delay was set at 4 s and a water pre-saturation pulse was applied during this period to cancel the water signal. The receiver gain was kept constant at a value of 90.5 to allow direct comparison between spectra. All experimental acquisition was automated and samples were held in the spectrometer for five minutes before data acquisition to allow temperature equilibration. The free induction decay (FID) values were multiplied by an exponential function equivalent to a 0.3Hz line-broadening factor before Fourier transformation. The resulting spectra were subject to automated phasing, to ensure all spectral peaks were directed upwards, and baseline correction, to ensure the baseline of all spectra was set to 0, using TopSpin 3.2 (Bruker Corporation, Germany). Spectra were imported into MatLab 2013 (MathWorks, Massachusetts, USA) using in-house scripts for all pre-processing steps. Spectra were calibrated to the α-glucose signal at 5.23ppm. For all spectra the region from 0.1-10ppm, to exclude the peak due to TSP, was divided into approximately 40,000 data points. The water signal region, 4.5-4.85ppm, was removed prior to further processing. All samples underwent probabilistic quotient (median fold) normalisation([Dieterle et al., 2006](#_ENREF_2)).

To aid metabolite identification further 2D experiments were carried out on selected samples. ^1^H-^1^H Correlation Spectroscopy (COSY), to demonstrate proton spins that are directly coupled to each other, and Total Correlation Spectroscopy (TOCSY), to demonstrate coupled protons up to 6 bonds apart within a molecule, experiments were performed. For COSY experiments a 12 ppm spectral width was used with a relaxation delay set at 1.2s during which a water pre-saturation pulse was applied to cancel the water signal. For TOCSY experiments a 12 ppm spectral width was used with a relaxation delay set at 2.0s during which a water pre-saturation pulse was applied to cancel the water signal.

**Analysis of variance with correction for false discovery rate of metabolic spectral data**

*Table 1. Univariate comparison of whole spectral data between those with pneumonia and those with brain injuries. Data are given as the metabolite associated with the spectral region analyzed, ppm, p-value and q-value following correction for false discovery rate. Spectral fragments with a q-value <0.1 are shown, significant regions were taken as those q<0.05.*

| ***Metabolite associated with spectral region*** | ***ppm*** | ***p-value*** | ***q-value*** |
| --- | --- | --- | --- |
| Cholesterol VLDL/HDL | 0.807 | 5.4 x10^-6^ | 0.009 |
| Cholesterol VLDL/HDL | 0.809 | 6.1 x10^-6^ | 0.009 |
| Cholesterol VLDL/HDL | 0.804 | 7.9x10 x10^-6^ | 0.009 |
| Cholesterol VLDL/HDL | 0.812 | 9.9 x10^-6^ | 0.009 |
| Cholesterol VLDL/HDL | 0.802 | 1.4 x10^-5^ | 0.010 |
| Cholesterol VLDL/HDL | 0.814 | 2.7 x10^-5^ | 0.017 |
| Alanine | 1.474 | 4.3 x10^-5^ | 0.022 |
| Cholesterol VLDL/HDL | 0.799 | 0.0001 | 0.022 |
| Lipids LDL/HDL | 1.204 | 0.0001 | 0.022 |
| Lipids LDL/HDL | 1.207 | 0.0001 | 0.022 |
| Cholesterol VLDL/HDL | 0.817 | 0.0001 | 0.031 |
| Phospholipids/choline | 3.187 | 0.0001 | 0.031 |
| Cholesterol VLDL/HDL | 0.797 | 0.0001 | 0.031 |
| Phospholipids/choline | 3.197 | 0.0001 | 0.031 |
| Alanine | 1.462 | 0.0001 | 0.031 |
| Phospholipids/choline | 3.194 | 0.0001 | 0.031 |
| Cholesterol VLDL/HDL | 0.794 | 0.0001 | 0.031 |
| Lipids LDL/HDL | 1.209 | 0.0002 | 0.031 |
| Phospholipids/choline | 3.189 | 0.0002 | 0.031 |
| Phospholipids/choline | 3.177 | 0.0002 | 0.031 |
| Alanine | 1.467 | 0.0002 | 0.031 |
| Phospholipids/choline | 3.199 | 0.0002 | 0.034 |
| Phenylalanine | 7.413 | 0.0002 | 0.034 |
| Alanine | 1.477 | 0.0003 | 0.037 |
| Phospholipids/choline | 3.179 | 0.0003 | 0.037 |
| Alanine | 1.479 | 0.0003 | 0.037 |
| Phenylalanine | 7.425 | 0.0003 | 0.037 |
| Cholesterol VLDL/HDL | 0.819 | 0.0004 | 0.048 |
| Phenylalanine | 7.428 | 0.0004 | 0.057 |
| Lipids LDL/HDL | 1.202 | 0.0005 | 0.058 |
| Phenylalanine | 7.415 | 0.0005 | 0.060 |
| Cholesterol VLDL/HDL | 0.792 | 0.0006 | 0.064 |
| Unidentified | 3.572 | 0.0006 | 0.064 |
| Phospholipids/choline | 3.184 | 0.0006 | 0.064 |
| Alanine | 1.464 | 0.0007 | 0.071 |
| Phospholipids/choline | 3.202 | 0.0007 | 0.072 |
| Glutamine | 2.474 | 0.0008 | 0.076 |
| Cholesterol VLDL/HDL | 0.787 | 0.0008 | 0.076 |
| Unidentified | 6.935 | 0.0009 | 0.082 |
| Cholesterol VLDL/HDL | 0.789 | 0.0009 | 0.082 |
| Unidentified | 2.944 | 0.0009 | 0.082 |
| Lipids LDL/HDL | 1.212 | 0.0010 | 0.084 |
| Glutamine | 2.489 | 0.0010 | 0.086 |
| Unidentified | 7.798 | 0.0010 | 0.087 |
| Unidentified | 2.917 | 0.0011 | 0.091 |
| Lipids LDL/HDL | 1.194 | 0.0011 | 0.091 |
| Unidentified | 2.932 | 0.0012 | 0.092 |
| Phospholipids/choline | 3.192 | 0.0012 | 0.092 |

*Table 2. Univariate comparison of whole spectral data between those with VAP and those with brain injuries. Data are given as the metabolite associated with the spectral region analyzed, ppm, p-value and q-value following correction for false discovery rate. The spectral fragments with the lowest 20 q-values are shown, significant regions were taken as those q<0.05.*

| ***Metabolite associated with spectral region*** | ***ppm*** | ***p-value*** | ***q-value*** |
| --- | --- | --- | --- |
| O-Glycoproteins | 2.062 | 0.0001 | 0.136 |
| O-Glycoproteins | 2.059 | 0.0001 | 0.136 |
| Phenylalanine | 7.423 | 0.0001 | 0.163 |
| Phenylalanine | 7.410 | 0.0002 | 0.163 |
| Unidentified | 2.924 | 0.0003 | 0.163 |
| O-Glycoproteins | 2.072 | 0.0003 | 0.163 |
| Phenylalanine | 7.333 | 0.0003 | 0.172 |
| O-Glycoproteins | 2.074 | 0.0004 | 0.181 |
| Unidentified | 2.927 | 0.0004 | 0.181 |
| N-Glycoproteins | 2.049 | 0.0006 | 0.198 |
| N-Glycoproteins | 2.052 | 0.0007 | 0.198 |
| O-Glycoproteins | 2.069 | 0.0007 | 0.198 |
| Unidentified | 5.165 | 0.0007 | 0.198 |
| O-Glycoproteins | 2.077 | 0.0008 | 0.198 |
| O-Glycoproteins | 2.064 | 0.0008 | 0.198 |
| Phenylalanine | 7.320 | 0.0010 | 0.221 |
| Unidentified | 5.163 | 0.0010 | 0.221 |
| N-Glycoproteins | 2.037 | 0.0011 | 0.221 |
| O-Glycoproteins | 2.057 | 0.0011 | 0.221 |
| N-Glycoproteins | 2.039 | 0.0013 | 0.240 |

**Multivariate Analysis of Lipid Data**

Figure 1. PCA scores plot (*R^2^X*=0·83 *Q^2^*=0·67) showing the first and second components, from a four component model, comparing samples taken at the first time point from patients admitted with brain injuries (blue circles) to those with pneumonia (red squares).

**

Figure 2. a) OPLS-DA scores plot, with one component (*R^2^Y*=0·31, *Q^2^Y*=0·19, p=0·02) comparing samples taken at the first time point from patients admitted with brain injuries (blue bars) to those with pneumonia (red bars). b) Loadings plot for the OPLS-DA model, lipid species directed upwards are more abundant in those with pneumonia and those directed downwards are more abundant in those with brain injuries. c) Table showing mean±standard deviation concentration for lipids with a q-values<0.1. Relative concentrations are given as pneumonia compared to brain injury. Lipids denoted by * are most important in causing discrimination in the OPLS-DA model. P- and q-values are given for ANOVA with correction for false discovery rate.

**

**a.**


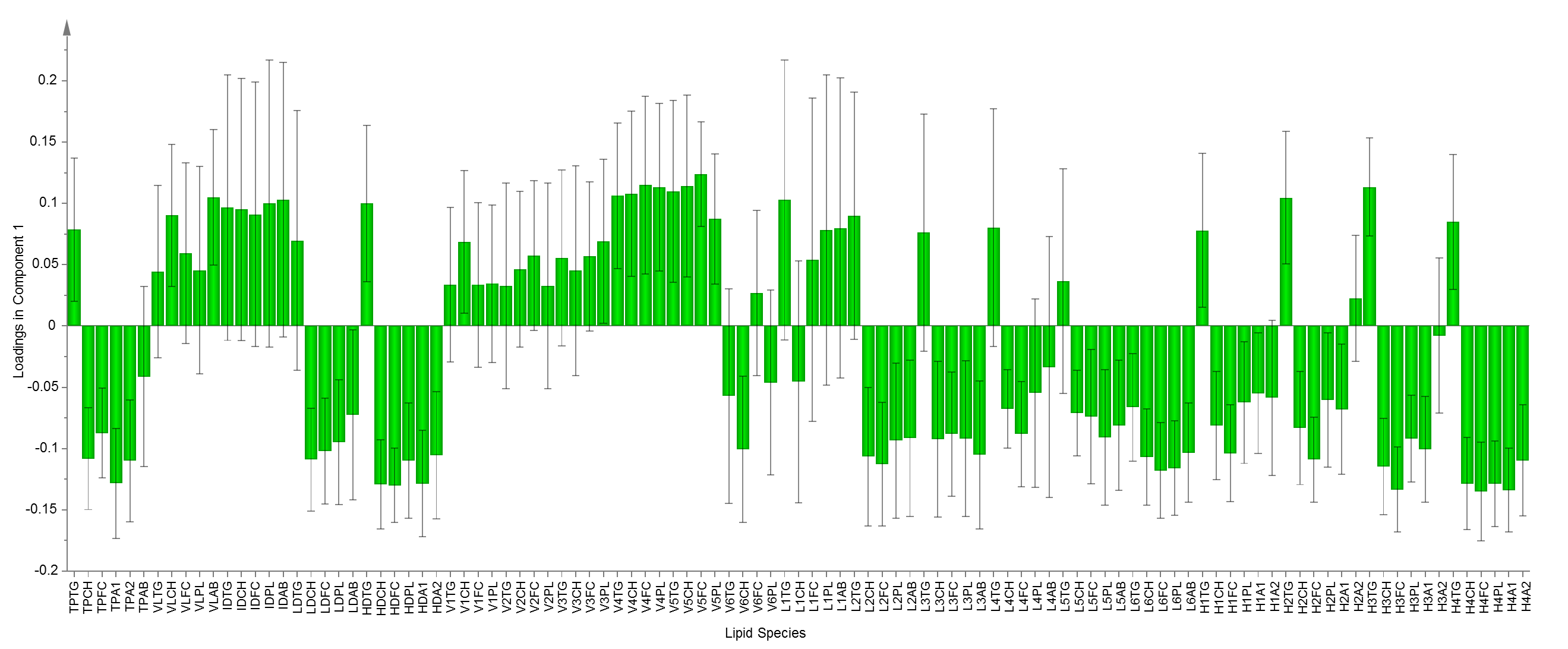


**b.**

| **Lipid Species**  **c.** | **Brain Injury** | **Pneumonia** | **Relative concentration in Pneumonia** | **p-value** | **q-value** |
| --- | --- | --- | --- | --- | --- |
| N | 26 | 15 | - | - | - |
| *H4FC in mg/dL | 5.5±2.1 | 2.3±2.2 | -2.36 | 4.2 x10^-5^ | 0.003 |
| *H4A1 in mg/dL | 84.8±24.1 | 46.8±29.3 | -1.81 | 0.0001 | 0.003 |
| *H4CH in mg/dL | 21.7±7.9 | 9.8±10.4 | -2.22 | 0.0002 | 0.006 |
| *H4PL in mg/dL | 30.1±9.8 | 16.9±11.4 | -1.78 | 0.0004 | 0.009 |
| *V5FC in mg/dL | 0.5±0.3 | 1.0±0.5 | 1.91 | 0.0009 | 0.020 |
| *H4A2 in mg/dL | 21.8±6.9 | 12.9±9.4 | -1.69 | 0.0013 | 0.020 |
| *H3FC in mg/dL | 3.9±1.3 | 2.4±1.4 | -1.60 | 0.0014 | 0.020 |
| *L2TG in mg/dL | 2.9±1.9 | 6.1±4.4 | 2.09 | 0.0024 | 0.031 |
| *L6FC in mg/dL | 9.2±5.4 | 4.1±4.0 | -2.23 | 0.0031 | 0.033 |
| *TPA2 in mg/dL | 45.6±8.1 | 35.8±11.9 | -1.27 | 0.0032 | 0.033 |
| *H1TG in mg/dL | 6.1±2.8 | 9.8±5.0 | 1.61 | 0.0036 | 0.033 |
| H3CH in mg/dL | 15.3±3.6 | 11.5±4.1 | -1.32 | 0.0038 | 0.033 |
| L1TG in mg/dL | 6.0±4.4 | 15.4±14.9 | 2.59 | 0.0044 | 0.035 |
| L5FC in mg/dL | 4.7±2.6 | 2.2±2.4 | -2.09 | 0.0049 | 0.035 |
| HDA2 in mg/dL | 46.4±7.7 | 37.5±11.5 | -1.24 | 0.0050 | 0.035 |
| L1AB in mg/dL | 4.6±4.0 | 11.1±10.1 | 2.40 | 0.0059 | 0.036 |
| L6CH in mg/dL | 33.4±21.2 | 15.4±14.6 | -2.17 | 0.0059 | 0.036 |
| H2TG in mg/dL | 3.0±1.3 | 4.6±2.3 | 1.53 | 0.0068 | 0.036 |
| L6AB in mg/dL | 20.0±12.5 | 9.6±8.6 | -2.08 | 0.0072 | 0.036 |
| L3TG in mg/dL | 3.0±1.9 | 5.7±4.3 | 1.92 | 0.0073 | 0.036 |
| L4TG in mg/dL | 2.8±2.0 | 6.3±5.7 | 2.23 | 0.0075 | 0.036 |
| HDCH in mg/dL | 78.6±24.5 | 54.4±29.9 | -1.45 | 0.0076 | 0.036 |
| L1PL in mg/dL | 5.0±5.1 | 12.6±12.4 | 2.55 | 0.0081 | 0.037 |
| HDTG in mg/dL | 14.9±6.1 | 22.0±10.3 | 1.48 | 0.0084 | 0.037 |
| L6PL in mg/dL | 18.8±10.4 | 10.1±8.4 | -1.86 | 0.0090 | 0.038 |
| TPA1 in mg/dL | 190.7±45.9 | 144.0±62.8 | -1.32 | 0.0093 | 0.038 |
| HDA1 in mg/dL | 191.5±49.3 | 144.4±64.2 | -1.33 | 0.0120 | 0.047 |
| H3A1 in mg/dL | 35.5±7.6 | 28.6±9.4 | -1.24 | 0.0139 | 0.052 |
| L5CH in mg/dL | 18.3±10.3 | 10.0±10.2 | -1.84 | 0.0162 | 0.058 |
| HDFC in mg/dL | 20.8±7.4 | 14.6±8.1 | -1.43 | 0.0166 | 0.058 |
| LDTG in mg/dL | 30.1±13.1 | 46.5±30.5 | 1.55 | 0.0212 | 0.072 |
| V5CH in mg/dL | 0.9±1.0 | 2.0±2.0 | 2.14 | 0.0276 | 0.091 |
| IDTG in mg/dL | 17.4±8.1 | 27.0±19.0 | 1.55 | 0.0298 | 0.095 |
| IDAB in mg/dL | 3.5±2.5 | 6.5±6.0 | 1.85 | 0.0311 | 0.096 |

Figure 3. PCA scores plot (*R^2^X*=0.75 *Q^2^*=0·56) showing the first and second components, from a three component model, comparing samples taken at the first time point from patients admitted with brain injuries (blue circles) to those with VAP (red triangles).

Figure 4. a) OPLS-DA scores plot, with one orthogonal and one aligned component (*R^2^Y=*0·63, *Q^2^Y*=0·47, p<0·01) comparing samples taken at the first time point from patients admitted with brain injuries (blue circles) to those with VAP (red triangles). b) Loadings plot for the OPLS-DA model; lipid species at the extremes of the x-axis represent those causing most discrimination between brain injury and VAP. c) Table showing mean±standard deviation concentration for lipids with a q-values<0.1. Relative concertation is given as VAP compared to brain injury. Lipids denoted by * are most important in causing discrimination in the OPLS-DA model. P- and q-values are given for ANOVA with correction for false discovery rate.

**a.**


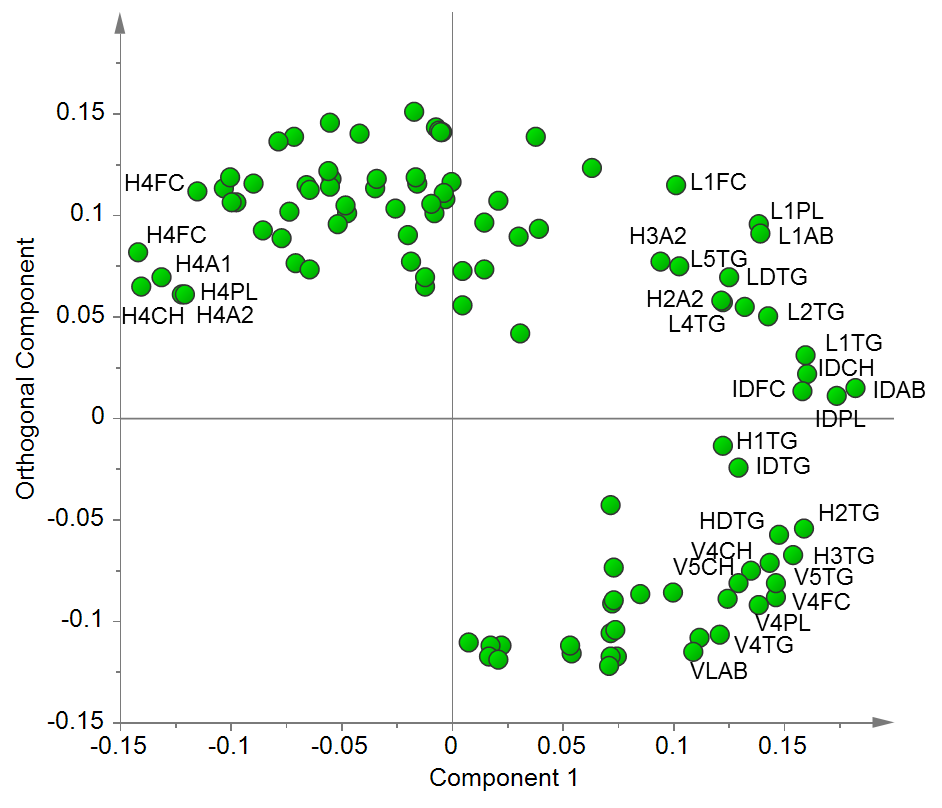


**b.**

| **Lipid Species**  **c.** | **Brain Injury** | **VAP** | **Relative concentration in VAP** | **p-value** | **q-value** |
| --- | --- | --- | --- | --- | --- |
| N | 26 | 7 | - | - | - |
| *IDCH in mg/dL | 9.7±7.2 | 26.9±6.5 | 2.77 | 3.1 x10^-6^ | 0.0003 |
| *IDAB in mg/dL | 3.5±2.5 | 8.7±2.0 | 2.48 | 1.6 x10^-5^ | 0.001 |
| *IDPL in mg/dL | 6.3±4.2 | 14.8±4.2 | 2.35 | 4.4 x10^-5^ | 0.002 |
| *H2TG in mg/dL | 3.0±1.3 | 5.3±1.2 | 1.76 | 0.0002 | 0.005 |
| *H3TG in mg/dL | 2.7±1.3 | 5.0±1.3 | 1.84 | 0.0002 | 0.005 |
| *IDFC in mg/dL | 2.1±2.2 | 5.8±2.0 | 2.70 | 0.0004 | 0.007 |
| *V5CH in mg/dL | 0.9±1.0 | 3.0±2.0 | 3.20 | 0.0006 | 0.007 |
| *L1TG in mg/dL | 6.0±4.4 | 13.5±5.5 | 2.26 | 0.0006 | 0.007 |
| *V4FC in mg/dL | 2.3±2.0 | 5.8±2.8 | 2.50 | 0.0007 | 0.007 |
| *V5TG in mg/dL | 3.2±1.6 | 5.9±1.9 | 1.83 | 0.0008 | 0.007 |
| *H4CH in mg/dL | 21.7±7.9 | 9.2±7.7 | -2.36 | 0.0008 | 0.007 |
| *H4FC in mg/dL | 5.5±2.1 | 2.3±2.0 | -2.37 | 0.0010 | 0.009 |
| *HDTG in mg/dL | 14.9±6.1 | 23.9±5.1 | 1.61 | 0.0012 | 0.009 |
| *V4CH in mg/dL | 4.8±4.1 | 11.7±6.0 | 2.43 | 0.0013 | 0.009 |
| *HDFC in mg/dL | 20.8±7.4 | 10.5±4.9 | -1.97 | 0.0016 | 0.011 |
| V4PL in mg/dL | 5.2±3.7 | 11.0±5.1 | 2.11 | 0.0019 | 0.012 |
| L4TG in mg/dL | 2.8±2.0 | 6.1±3.4 | 2.18 | 0.0020 | 0.012 |
| V5PL in mg/dL | 1.1±1.1 | 3.1±2.0 | 2.70 | 0.0022 | 0.013 |
| HDCH in mg/dL | 78.6±24.5 | 46.1±15.3 | -1.71 | 0.0023 | 0.013 |
| H4A1 in mg/dL | 84.8±24.1 | 50.9±24.7 | -1.67 | 0.0025 | 0.013 |
| L1AB in mg/dL | 4.6±4.0 | 10.1±4.4 | 2.18 | 0.0037 | 0.018 |
| L1PL in mg/dL | 5.0±5.1 | 11.7±4.9 | 2.36 | 0.0039 | 0.018 |
| L2TG in mg/dL | 2.9±1.9 | 5.5±2.6 | 1.88 | 0.0056 | 0.025 |
| HDA1 in mg/dL | 191.5±49.3 | 134.9±32.2 | -1.42 | 0.0075 | 0.032 |
| TPA1 in mg/dL | 190.7±45.9 | 138.0±30.5 | -1.38 | 0.0076 | 0.032 |
| H4PL in mg/dL | 30.1±9.8 | 18.5±9.4 | -1.62 | 0.0089 | 0.036 |
| H1FC in mg/dL | 8.5±4.1 | 4.0±2.0 | -2.14 | 0.0093 | 0.036 |
| IDTG in mg/dL | 17.4±8.1 | 27.2±10.3 | 1.56 | 0.0115 | 0.043 |
| H2A2 in mg/dL | 6.3±1.6 | 8.2±1.7 | 1.29 | 0.0125 | 0.045 |
| H1CH in mg/dL | 27.4±15.3 | 11.8±6.3 | -2.33 | 0.0135 | 0.046 |
| LDTG in mg/dL | 30.1±13.1 | 45.0±14.7 | 1.50 | 0.0136 | 0.046 |
| H4A2 in mg/dL | 21.8±6.9 | 14.3±6.4 | -1.53 | 0.0141 | 0.046 |
| L3TG in mg/dL | 3.0±1.9 | 5.2±2.5 | 1.75 | 0.0151 | 0.047 |
| V4TG in mg/dL | 12.0±8.3 | 21.4±9.8 | 1.79 | 0.0153 | 0.047 |
| V5FC in mg/dL | 0.5±0.3 | 0.9±0.3 | 1.71 | 0.0199 | 0.060 |
| L6FC in mg/dL | 9.2±5.4 | 4.2±2.4 | -2.21 | 0.0241 | 0.070 |
| H1TG in mg/dL | 6.1±2.8 | 8.7±2.0 | 1.44 | 0.0251 | 0.071 |
| H3A2 in mg/dL | 10.1±1.7 | 11.8±1.9 | 1.17 | 0.0281 | 0.078 |
| H4TG in mg/dL | 2.8±1.5 | 4.2±1.4 | 1.52 | 0.0331 | 0.088 |
| L2FC in mg/dL | 4.9±4.1 | 1.3±1.4 | -3.68 | 0.0336 | 0.088 |
| VLAB in mg/dL | 6.4±4.7 | 10.8±4.9 | 1.68 | 0.0378 | 0.097 |
| VLCH in mg/dL | 16.1±14.9 | 29.9±15.7 | 1.86 | 0.0388 | 0.097 |
| L6PL in mg/dL | 18.8±10.4 | 10.1±4.5 | -1.86 | 0.0404 | 0.099 |
| L5TG in mg/dL | 3.0±2.0 | 4.8±2.0 | 1.61 | 0.0413 | 0.099 |

**References**

Beckonert, O., Keun, H. C., Ebbels, T. M., Bundy, J., Holmes, E., Lindon, J. C. & Nicholson, J. K. Metabolic profiling, metabolomic and metabonomic procedures for NMR spectroscopy of urine, plasma, serum and tissue extracts. *Nat Protoc,* 2007; 2**,** 2692-703.

Dieterle, F., Ross, A., Schlotterbeck, G. & Senn, H. Probabilistic quotient normalization as robust method to account for dilution of complex biological mixtures. Application in 1H NMR metabonomics. *Anal Chem,* 2006; 78**,** 4281-90.

Dona, A. C., Jimenez, B., Schafer, H., Humpfer, E., Spraul, M., Lewis, M. R., Pearce, J. T., Holmes, E., Lindon, J. C. & Nicholson, J. K. Precision high-throughput proton NMR spectroscopy of human urine, serum, and plasma for large-scale metabolic phenotyping. *Anal Chem,* 2014; 86**,** 9887-94.
